# Isometric Conditioning Activity and Jump Performance: Impact of Training Status in Male Participants

**DOI:** 10.3390/jcm14176214

**Published:** 2025-09-03

**Authors:** Jakub Jarosz, Andrzej Szwarc

**Affiliations:** 1 Institute of Sport Sciences, Academy of Physical Education, 40-065 Katowice, Poland; 2 Department of Team Sport Games, Gdansk University of Physical Education and Sport, 80-336 Gdansk, Poland; andrzej.szwarc@awf.gda.pl

**Keywords:** post-activation performance enhancement, countermovement jump, neuromuscular potentiation, trained participants, highly trained participants

## Abstract

**Background/Objectives:** Post-activation performance enhancement (PAPE) is an acute neuromuscular phenomenon influenced by training status, yet evidence regarding its response to isometric conditioning activity (ICA) across different athletic populations remains inconclusive. This study investigated the acute effects of ICA on countermovement jump (CMJ) performance in trained (T) versus highly trained (HT) male participants. **Methods:** A total of 32 participants (T: *n* = 16; HT: *n* = 16) completed two randomized sessions: a control condition (CTRL) and an isometric protocol (ICA; three sets of three maximal isometric back squat contractions, 3 s each). CMJ height was assessed at baseline and at 3-, 6- and 9-min post-intervention using a force platform. Repeated-measures ANOVA examined interactions between time, condition, and training status. **Results:** A significant improvement in jump height was observed only in the HT-ISO group at 3 min post-ICA (mean difference: +3.0 ± 2.3 cm; *p* < 0.005; *d* = 0.65). No significant changes were detected in the T group across conditions. Peak power and modified reactive strength index showed no significant differences, though effect trends favored the HT group. **Conclusions:** ICA elicits short-term PAPE effects in highly trained, but not moderately trained, individuals. These findings underscore the importance of tailoring warm-up protocols to the athlete’s training level for optimal performance enhancement.

## 1. Introduction

Post-activation performance enhancement (PAPE) is a neuromuscular phenomenon characterized by acute improvements in performance following the execution of specific high-intensity conditioning activities (CAs) [1,2]. Isometric conditioning activities (ICAs) for example, isometric squats executed via a pushing isometric muscle action (PIMA) [3]—are among the most frequently employed approaches, alongside isotonic protocols, for eliciting the PAPE effect. These ICAs are usually implemented immediately before high-intensity athletic movements that share comparable biomechanical demands; for instance, performing a back squat prior to vertical jump tasks [4,5,6]. The PAPE phenomenon arises from the fact that the potentiation effect is predominantly local [1,2] and can be explained by alterations within the engaged musculature, including phosphorylation of myosin regulatory light chains, elevated muscle temperature, decreased intramuscular pH leading to increased hydrogen ion concentration, enhanced blood flow, increased intracellular water content, augmented neuromuscular activation, and greater stiffness of the muscle–tendon unit [6,7,8]. Furthermore, studies by Wilson et al. [6] and Suchomel et al. [7] indicate that training status can modulate the balance between potentiation and fatigue, which may, in turn, influence the magnitude of the PAPE effect following ICA.

The ICA has garnered increasing attention in both scientific research and practical sports applications owing chiefly to its straightforward implementation and adaptability to a variety of training regimens without the need for specialized equipment [9,10]. Nonetheless, the scientific literature remains inconclusive regarding the effects of ICA on the development of explosive power [9,10,11,12,13,14,15,16,17,18,19,20,21]. Conversely, other reports, including those by Jarosz et al. [10], French et al. [13], and Krzysztofik et al. [19], fail to demonstrate a beneficial impact of ICA on CMJ performance, despite the use of identical activation exercises. The PAPE effect elicited by ICA is influenced by multiple factors, including the knee joint angle during contraction [12], contraction intensity [22], total duration and number of ICA sets [9], and contraction distribution strategy [10]. A key yet underexplored moderator of the ICA-induced PAPE response is the training status of participants. Precise classification of training status is essential for valid interpretation and generalization of results [23]. To address this issue, McKay et al. [23] proposed a standardized six-tier classification system for athletic populations, based on training volume and performance metrics. This framework categorizes individuals as follows: Tier 0—Sedentary; Tier 1—Recreationally Active; Tier 2—Trained/Developmental; Tier 3—Highly Trained/National Level; Tier 4—Elite/International Level; and Tier 5—World Class. In a recent meta-analysis, Xu et al. [24] demonstrated that the PAPE effect, when compared to a control condition (CTRL), was more pronounced in highly trained individuals (effect size [ES] = 0.38) than in trained (ES = 0.21) or recreationally active individuals (ES = 0.22). However, previous meta-analyses have generally assessed the overall impact of CAs without distinguishing between different CA modalities, namely isotonic, isometric, or plyometric. This distinction is critical, as ICA may produce unique effects compared to traditional isotonic CAs, due to lower muscle damage, reduced fatigue, and potentially distinct neuromuscular mechanisms of action [25]. Thus, findings derived from isotonic or plyometric CA protocols may not be directly applicable to ICA [9]. Furthermore, most prior analyses have primarily focused on comparisons between individual training-level groups and CTRL condition, which limits the understanding of more nuanced relationships between adjacent training status levels. Direct comparisons between neighboring categories (e.g., trained vs. highly trained) may yield more refined insights into how training status modulates the PAPE response. Despite the increasing scientific interest in individual factors influencing the PAPE effect, to the best of the authors’ knowledge, no previous studies have directly compared the acute effects of ICA on performance between trained and highly trained participants. Such investigations may provide valuable insights into optimizing motor preparation strategies based on athletes’ training status and contribute to the more precise adjustment of ICA protocols in practical training settings.

The aim of the present study was therefore to address this gap by examining the influence of training status on countermovement jump (CMJ) performance following an isometric protocol—ICA = three sets of three isometric contractions lasting 3 s each (ISO), relative to a CTRL condition. CMJ height was measured at 3-, 6-, and 9-min post-ICA. We hypothesized that both trained (T) and highly trained (HT) participants would demonstrate significant increases in CMJ height across at all post-intervention time points, with the largest mean improvement occurring at 6 min post-ICA, corresponding to the typical peak of the PAPE response [1]. Furthermore, we specifically predicted the HT group would exhibit greater relative gains in CMJ height than the T group, reflecting a stronger PAPE effect in more highly trained athletes.

## 2. Materials and Methods

### 2.1. Experimental Approach to the Problem

A randomized, single-blind, parallel-group interventional study was conducted, in which each participant took part in two experimental sessions. The aim was to compare the acute effects of a maximal isometric squat, performed as an ICA, on CMJ height, depending on the participants’ training status, i.e., T and HT.

Participants were randomly assigned to two different experimental conditions.

1. CTRL—maintaining light physical activity, where participants were to walk on a treadmill at a speed of 5 km/h for the time equivalent to performing the entire ICA set (9 min).
2. ISO—3 sets of ICA, each consisting of 3 repetitions of 3 s maximal isometric contractions, with a total ICA duration of 1 set per 9 s = 27 s, including 3 min rest intervals between sets.

**Figure 1:**
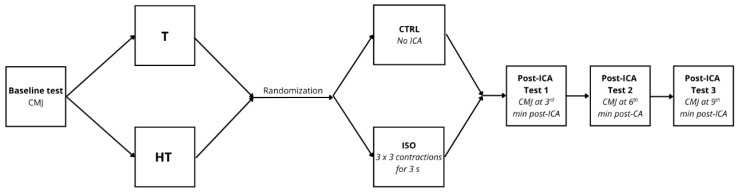
Study design flowchart. T—group consisting of trained participants; HT—group consisting of highly trained participants; CMJ—countermovement jump; ICA—isometric conditioning activity; CTRL—control condition (without ICA); ISO—isometric muscle action condition with total ICA duration of 27 s in 3 sets.

The CMJ measurements were taken approximately 3 min before the CA, and 3-, 6-, and 9-min post-ICA [8]. In the CTRL condition, measurements were taken at the same time points but without the application of ICA (Figure 1). All sessions were separated by 4–7 days.

### 2.2. Participants

The sample size was determined using G*Power version 3.1.9.2 (Dusseldorf, Germany), with the following parameters for the statistical test: ANOVA for repeated measures with a within-factors (two group of participants, two experimental conditions, and four measurements), a statistical power of 0.8, a significance level of 0.05, and an effect size of approximately d = 0.5 based on previous studies evaluating the immediate impact of isometric activation exercises on jump performance [15,19]. The analysis indicated that the minimum required sample size for this study was 24 participants.

The study involved 32 male participants, comprising 16 trained participants (aged 20–26 years) and 16 highly trained volleyball participants (aged 20–33 years). Participants were classified according to their training status and athletic level, based on the criteria established by McKay et al. [23] (Table 1).

The inclusion criteria for the trained group were as follows:

(a) Resistance training frequency of ≥3 sessions per week for a minimum of 2 years;
(b) Squat relative 1-repetition maximum (1RM) ≥ 1.2 × body mass;
(c) No history of musculoskeletal injuries resulting in training absence exceeding 4 weeks within 6 months prior to the commencement of the study.

The inclusion criteria for the highly trained group were as follows:

(a) Volleyball training experience of ≥5 years;
(b) Resistance training frequency of ≥3 sessions per week continuously for at least 5 years;
(c) Squat relative 1-repetition maximum (1RM) ≥ 1.4 × body mass;
(d) No musculoskeletal injuries resulting in a training interruption longer than 4 weeks within 6 months preceding the study.

**Figure 2:**
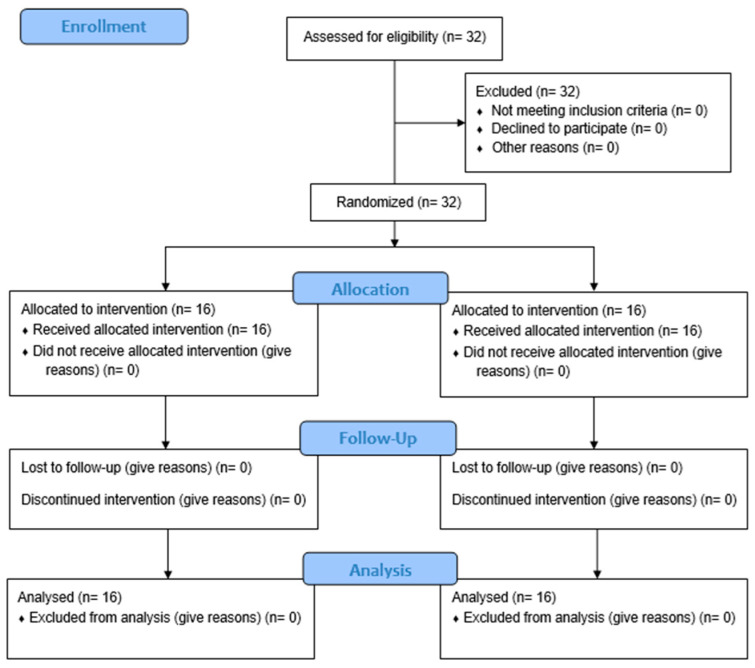
Participant flow diagram.

All participants were instructed to maintain their habitual dietary patterns and to refrain from using any supplements or stimulants, except for routine supplementation (e.g., creatine), during the week preceding the experiment. During the experimental session, body composition was assessed using bioelectrical impedance analysis with the InBody 770 device (InBody 770, Biospace Co., Ltd., Seoul, Republic of Korea), under controlled laboratory conditions. The study employed a randomized crossover design with participant blinding regarding the specific experimental condition to which they were assigned. Randomization was conducted using an online randomization tool (randomization.org)which assigned each participant a unique identifier and session sequence. Following allocation, participants were not informed of subsequent procedures [9]. Detailed information regarding the study’s purpose and expected outcomes was also withheld to minimize bias. All participants were informed about the potential risks and benefits of the study, as well as their right to withdraw at any point without providing justification. Written informed consent was obtained from all participants, although specific details concerning the study objectives and anticipated findings were not disclosed. All participants completed the experiment (Figure 2). The entire research protocol was conducted at the Academy of Physical Education in Katowice, Poland. The experiment received formal approval from the Bioethics Committee for Scientific Research (03/2021, approval date 27 May 2021) at the Academy of Physical Education in Katowice in accordance with the ethical principles outlined in the Declaration of Helsinki of 1983.

The following Table 1 details the descriptive characteristics of the trained and highly trained participant cohorts.

### 2.3. Procedure

Each testing session commenced with a standardized warm-up protocol comprising 5 min of cycling on a stationary ergometer, followed by a sequence of dynamic exercises including bodyweight squats (10 repetitions), forward lunges (10 repetitions), leg swings (10 repetitions), jumping jacks (10 repetitions), and CMJs (5 repetitions). Following the warm-up, participants performed a baseline assessment of CMJ height. Baseline variability of CMJ performance between sessions was not assessed in the present study. After a rest interval of approximately 3 min, participants underwent either the ICA or no ICA (CTRL), with the order of conditions counterbalanced across participants via randomization. During the ICA, participants were positioned under a fixed and immobile barbell, securely placed across the upper trapezius. Squat depth was standardized using a knee joint angle of 120 degrees [9,10], as determined and confirmed by a trained strength and conditioning specialist using a goniometer (EasyAngle, Meloq AB, Stockholm, Sweden). Body alignment was standardized across all trials by a certified weightlifting coach to maintain an upright torso position. Upon receiving a verbal command from the researcher, participants were instructed to “push the barbell vertically upward as hard and as fast as possible” while maintaining full-body tension, bracing their backs against the bar, and exerting maximal force through their feet into the ground. To ensure maximal effort during each isometric contraction attempt, participants were strongly verbally encouraged throughout the trial, following common procedures used in neuromuscular testing [26]. During subsequent CMJ trials, the depth of the countermovement phase was not constrained, allowing participants to self-select their preferred range of motion.

### 2.4. Measurement of Countermovement Jump Performance

Jump performance was evaluated utilizing a force platform (Force Decks, Vald Performance, Australia), operating at a sampling frequency of 1000 Hz. This tool is widely recognized for its validity and reliability in assessing the kinematics of vertical jumps [27]. The vertical ground reaction forces were subsequently processed and analyzed using a dedicated software (https://valdperformance.com/forcedecks/, 21 July 2025), ensuring standardized data acquisition and computation procedures. Each subject performed three CMJs without the use of arm swing, with a standardized 5 s rest interval between trials. Participants initiated each trial from an upright standing posture with hands placed on the iliac crests to minimize extraneous motion and ensure postural neutrality, thereby reducing angular displacement at the hip joint. Prior to initiating the countermovement phase, individuals were instructed to maintain static equilibrium for a minimum of one second. Subsequently, they executed a countermovement to a self-determined depth, immediately followed by a maximal-effort vertical propulsion. Subjects were required to land in the original position, centrally on the force platform. All measurements were conducted by the same experienced technician to ensure consistency. For further analysis, the best trial (i.e., the one with the highest jump height) out of the three attempts was used. The primary outcome measure was jump height (JH), computed based on the vertical velocity of the center of mass at takeoff, derived using the impulse–momentum relationship. Additional variables including relative peak power output (PP) and modified reactive strength index (RSImod) were considered as factors potentially influencing jump height.

### 2.5. Statistical Analyses

All statistical analyses were conducted using JASP (version 0.18.3; JASP Team, University of Amsterdam, The Netherlands) and are presented as means with standard deviations (±SD) (Table 2). Statistical significance was set at *p* < 0.05. A preliminary step aimed at approximating the empirical distribution to normality was performed. Outliers were then detected using the interquartile range (IQR), defined as Q3 − Q1. Data points below Q1 − 1.5 × IQR or above Q3 + 1.5 × IQR were flagged and reviewed; a single genuine extremum was retained for correction. Winsorization was applied by clipping values below the 5th percentile up to that threshold and reducing values above the 95th percentile down to that threshold [28]. For the variables RSImod: T-CTRL (baseline), T-ISO (9th post-ICA), H-CTRL (3rd post-ICA), and HT-ISO (baseline and 9th post-ICA), only one outlier—the single observation—exceeded the 95th percentile and was clipped. The Shapiro–Wilk test was used to assess the normality of the data distribution. Homogeneity of variances was verified using Levene’s test. Mauchly’s test of sphericity was applied to assess the assumption of sphericity in the repeated-measures design. The repeated-measures three-way ANOVA (2 groups [T and HT] × 2 conditions [CTRL and ISO] × 4 time points [pre-ICA; 3rd, 6th and 9th minute post-ICA]) were used to investigate the influence of ICA on CMJ selected variables. When a significant main effect or interaction was found, the post hoc tests with Bonferroni correction (*p_bonf*) were used to analyze pairwise comparisons. The order of conditions was counterbalanced across participants to minimize potential order effects. The effect sizes were determined by Cohen’s d, which was characterized as “trivial” (|*d*| < 0.20), “small” (0.20 ≤ |*d*| < 0.50), “moderate” (0.50 ≤ |*d*| < 0.80), or “large” (|*d*| ≥ 0.80) [29].

## 3. Results

The following Table 2 summarizes the alterations in JH, PP, and RSImod across successive measurement time points and experimental conditions.

The following Table 3 presents the ANOVA-derived p-values for the main effects of condition, time, and group, as well as their interaction terms, on JH, PP, and RSImod.

### 3.1. Jump Height

A repeated-measures three-way ANOVA revealed a significant multi-interaction for JH (*p* = 0.036; ηp^2^ = 0.009). Post hoc comparisons indicated a significant increase in JH in the HT-ISO condition at 3 min post-ICA compared to pre-ICA for JH (mean difference [MD] = 3 ± 2.3 cm; Cohen’s d = 0.65; *p_bonf* < 0.005) (Table 3).

### 3.2. Relative Peak Power

A repeated-measures three-way ANOVA revealed a significant interaction effect between condition and group (*p* = 0.006; ηp^2^ = 0.013). However, post hoc comparisons did not reveal any significant pairwise differences (all *p* > 0.05) (Table 3).

**Figure 3:**
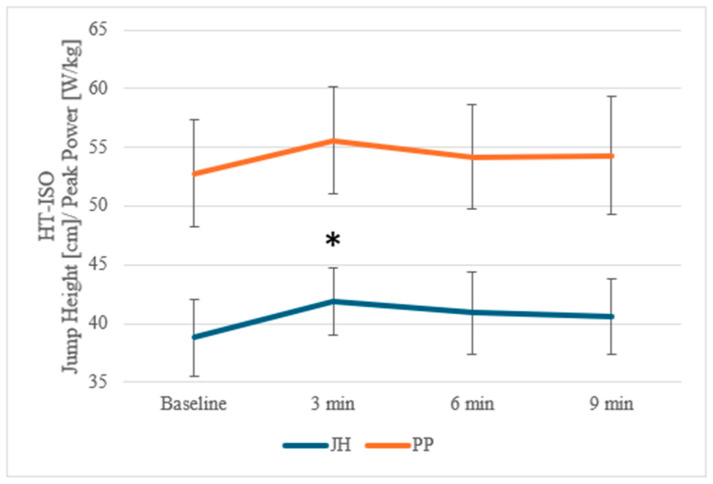
Changes in jump height (cm) and peak power (W/kg) over time—HT-ISO. HT-ISO—group consisting of highly trained participants with isometric muscle action condition with total ICA duration of 27 s in 3 sets; 3 min—3rd minute post-ICA, 6 min—6th minute post-ICA, 9 min—9th minute post-ICA; JH—jump height (cm); PP—peak power (W/kg); *—significant difference compared to baseline value and the 3rd-minute post-ICA within the same condition, *p* < 0.05.

For improved clarity of the results, changes in JH and PP over time for HT-ISO are presented in Figure 3.

**Figure 4:**
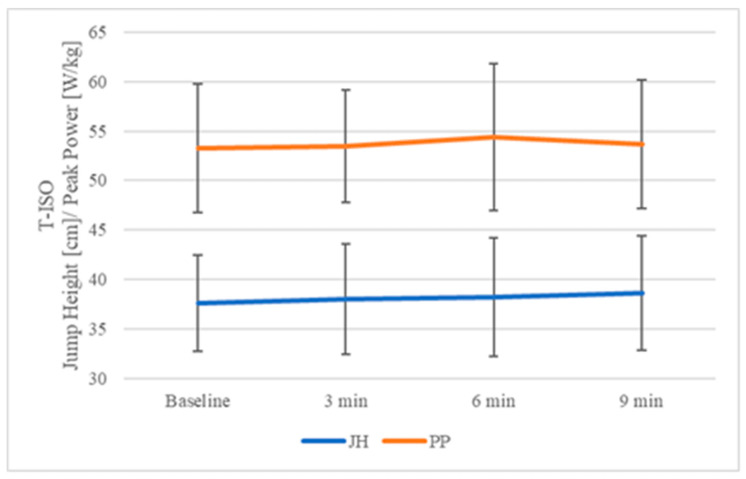
Changes in jump height (cm) and peak power (W/kg) over time—T-ISO. T-ISO—group consisting of trained participants with isometric muscle action condition with total ICA duration of 27 s in 3 sets; 3 min—3rd minute post-ICA; 6 min—6th minute post-ICA; 9 min—9th minute post-ICA; JH—jump height (cm); PP—peak power (W/kg).

For improved clarity of the results, changes in JH and PP over time for T-ISO are presented in Figure 4.

### 3.3. RSI Modified

A repeated-measures three-way ANOVA revealed a significant interaction effect between condition and group (*p* = 0.014; ηp^2^ = 0.022). However, post hoc comparisons did not reveal any significant pairwise differences (all *p* > 0.05) (Table 3).

## 4. Discussion

The results of the present study partially corroborate the proposed hypotheses. Contrary to the first hypothesis—which predicted that both training groups (T and HT) would demonstrate significant increases in CMJ height following the ISO protocol—a significant enhancement in JH was observed exclusively in the HT-ISO group, while no statistically meaningful improvement emerged in the T-ISO group. Moreover, contrary to the second hypothesis anticipating greater gains in HT than in T, particularly 6 min post-ICA, the HT group did exhibit elevated JH values at that time point; however, the peak effect occurred at 3 min (rather than 6 min) post-ICA. Moreover, regardless of training status and experimental condition, no significant differences were found in other neuromuscular parameters, including PP and RSImod. It is noteworthy that PP in the HT-ISO condition approached statistical significance, which is relevant for interpreting the findings. These results highlight that only highly trained athletes are capable of effectively utilizing the ISO protocol for short-term improvements in explosive performance.

The observed improvement in CMJ test performance following the implementation of the ISO protocol in the HT group is consistent with previous findings reported by Jarosz et al. [9] and Spieszny et al. [15], who also demonstrated a beneficial effect of the same ICA protocol on vertical jump parameters in HT athletes. Of particular significance was the statistically significant increase in CMJ height recorded as early as 3 min post-ICA application in the HT-ISO group. This result may indicate an optimal balance between potentiation and fatigue, corroborating earlier observations in the HT group by Jarosz et al. [9]. One of the potential mechanisms underlying these effects is the intense activation of motor units during isometric exercise, which results in enhanced recruitment of muscle fibers [30,31]. Moreover, isometric exercises are characterized by a relatively low energetic cost compared to eccentric–concentric activities, such as barbell squats. This allows for the execution of a conditioning stimulus while preserving a high level of muscle functional capacity within a short time after exertion [32,33,34]. However, caution should be exercised in interpreting these findings. Despite statistical significance, the observed effects were of moderate magnitude, and their practical relevance in competitive settings may be limited. Therefore, further research is warranted to determine the reliability and applicability of this effect across various contexts and athletic populations.

The organism’s response to PAPE protocols is highly dependent on individual athlete characteristics—such as biological age, training experience, and the specific demands of the sport practiced—which should be regarded not only as control variables but, more importantly, as moderators of the intervention’s effectiveness [2,23]. Consequently, PAPE protocols are not universal tools; rather, they require precise individualization, taking into account the athlete’s stage of athletic development and specific neuromuscular adaptations [2]. Although relative strength was similar between groups (T: 1.44 ± 0.2 vs. HT: 1.49 ± 0.3 kg/bm), prior research by Seitz and Haff [2] and Guerra et al. [35] suggests that absolute strength is a more accurate indicator of potential responsiveness to PAPE stimuli. The HT group comprised athletes specializing in explosive power sports (e.g., volleyball), with extensive strength training experience (9.3 ± 4.2 years), whereas the T group consisted of recreational or non-specialist trainees with considerably less experience (3.5 ± 1.2 years). The shorter training history in the T group likely contributed to less efficient motor unit recruitment and a weaker adaptive response to the applied isometric stimulus, in contrast to the HT group, which likely exhibited more favorable neuromuscular adaptations conducive to PAPE manifestation due to a higher level of neuromuscular conditioning. As noted by Seitz and Haff [2] and Hodgson et al. [36], these mechanisms may be related to increased phosphorylation of myosin regulatory light chains, enhanced motor unit recruitment, and greater tolerance to training-induced fatigue among highly trained individuals [6,7,36,37], whereas lower body mass, stature, and biological age among T group participants may have limited their capacity to generate absolute force, thereby constraining their neuromuscular performance. The consequence of reduced neuromuscular efficiency and limited experience in generating high muscular tension in the T group may have rendered the ICA protocol overly demanding, diminishing or nullifying the potential PAPE response in this cohort. These findings support the notion that the efficacy of PAPE interventions is strongly influenced by the balance between potentiation and fatigue [10]. In summary, while the ISO protocol may be effective, its efficacy appears to be limited to well-trained individuals with appropriate physiological characteristics.

However, the present study is not without limitations. The T group was heterogeneous in terms of the type and intensity of physical activities practiced, which may have contributed to variability in responses to the ICA protocol. Additionally, fatigue levels and relevant physiological parameters were not directly monitored. Techniques such as tensiomyography, surface electromyography, mechanical muscle property assessment [38], and isometric strength monitoring could have provided a more detailed understanding of the mechanisms underlying the observed effects. Future research should consider the use of more homogeneous participant groups to reduce inter-individual variability. Moreover, the lack of comprehensive group characterization—such as measurement of VO_2_max—represents an additional limitation of the present study. Studies with larger numbers of participants will also be essential to improve generalizability and deepen our understanding of these results. It would also be beneficial to include biochemical blood analyses (e.g., lactate, creatine kinase, and cortisol levels) in response to different types of activation (isometric, isotonic, and plyometric) and to examine their relationship with training status. Such an approach would allow for a better understanding of the interaction between fatigue and activation and would facilitate the more precise individualization of ICA protocols according to athletes’ specific needs.

## 5. Conclusions and Practical Implications

The results indicate that the effectiveness of the ICA protocol in eliciting the PAPE effect is closely linked to the athletes’ level of training. A significant increase in JH during the CMJ test at 3 min post-ICA was observed only in the group with a higher training level (HT-ISO), confirming the presence of the PAPE effect exclusively in this group. The absence of a similar response in the less trained group (T) suggests that a lower training status may limit the ability to exploit the potential benefits of the ICA protocol. This highlights the need to individualize ICA parameters—such as the number of sets, contraction duration, rest intervals, and intensity—based on the athlete’s profile. In this study, the three sets with three contractions per 3 s isometric squat protocol with 3 min rests was effective for trained individuals. For less trained athletes, reducing volume (e.g., to two sets) or increasing rest (e.g., to 4–5 min) may be more suitable to ensure safe and effective activation. Tailoring these parameters to the individual’s training capacity forms the basis for the successful application of PAPE strategies in both clinical and athletic practice.

## Figures and Tables

**Table 1:** Descriptive characteristics of trained and highly trained participants.

	T [*n* = 16]	HT [*n* = 16]
Age [years]	22 ± 2	27 ± 4
Body mass [kg]	78 ± 8	91 ± 13
Body fat [%]	10.2 ± 3.4	10 ± 2.2
Body height [cm]	180 ± 5	196 ± 8
Resistance training experience [years]	3.5 ± 1.2	9.3 ± 4.2
Relative of 1RM BS [kg/bm]	1.44 ± 0.2	1.49 ± 0.3

T—trained participants; HT—highly trained participants; 1RM—one-repetition maximum; BS—back squat; bm—body mass.

**Table 2:** Change in jump height, peak power, and RSImod between time points and conditions.

			Baseline	3 min	6 min	9 min
Jump Height [cm]	T	CTRL	38.9 ± 4.2	40.0 ± 4.9	39.5 ± 5.8	39.1 ± 5.3
		ISO	37.6 ± 4.9	38 ± 5.6	38.2 ± 6	38.6 ± 5.8
	HT	CTRL	39.4 ± 2.9	38.1 ± 3	39.5 ± 2.3	38.6 ± 2.5
		ISO	38.8 ± 3.3	41.9 ± 2.9	40.9 ± 3.5	40.6 ± 3.2
Peak Power [W/kg]	T	CTRL	54.5 ± 6.6	54.8 ± 6.7	53.9 ± 6.4	53.3 ± 6.7
		ISO	53.3 ± 6.5	53.5 ± 5.7	54.4 ± 7.4	53.7 ± 6.5
	HT	CTRL	52.5 ± 3.1	51.4 ± 3.9	52.2 ± 3.8	52.0 ± 3.4
		ISO	52.8 ± 4.6	55.6 ± 4.6	54.2 ± 4.4	54.3 ± 5
RSImod	T	CTRL	0.49 ± 0.10	0.51 ± 0.11	0.49 ± 0.09	0.47 ± 0.09
		ISO	0.47 ± 0.10	0.49 ± 0.09	0.48 ± 0.10	0.50 ± 0.11
	HT	CTRL	0.48 ± 0.04	0.46 ± 0.04	0.49 ± 0.05	0.48 ± 0.05
		ISO	0.51 ± 0.07	0.52 ± 0.06	0.51 ± 0.07	0.51 ± 0.07

CTRL—control condition (without ICA); ISO—condition with the 3 sets of ICA, each consisting of 3 repetitions of 3 s maximal isometric contractions; 3 min—3rd minute post-ICA; 6 min—6th minute post-ICA; 9 min—9th minute post-ICA; T—group consisting of trained participants; HT—group consisting of highly trained participants.

**Table 3:** ANOVA-derived p-values for main effects and interaction terms for jump height, peak power, and RSImod by condition, time, and group.

			Condition	Time	Group	Condition × Time	Condition × Group	Time × Group	Condition × Time × Group
Jump Height [cm]	T	CTRL	*p* = 0.193	*p* = 0.009 *	*p* = 0.262	*p* = 0.517	*p* = 0.379	*p* = 0.511	*p* = 0.036 *
		ISO							
	HT	CTRL							
		ISO							
Peak Power [W/kg]	T	CTRL	*p* = 0.264	*p* = 0.663	*p* = 0.711	*p* = 0.333	*p* = 0.006 *	*p* = 0.692	*p* = 0.287
		ISO							
	HT	CTRL							
		ISO							
RSImod	T	CTRL	*p* = 0.277	*p* = 0.264	*p* = 0.666	*p* = 0.593	*p* = 0.014 *	*p* = 0.203	*p* = 0.339
		ISO							
	HT	CTRL							
		ISO							

CTRL—control condition (without ICA); ISO— condition with the 3 sets of ICA, each consisting of 3 repetitions of 3 s maximal isometric contractions; T—group consisting of trained participants; HT—group consisting of highly trained participants; *—significant difference in comparison to baseline value within the condition, *p* < 0.05.

## Data Availability

The datasets analyzed during the current study are available from the corresponding author upon reasonable request.

## Abbreviations

The following abbreviations are used in this manuscript:

CA	Conditioning activity
ICA	Isometric conditioning activity
PAPE	Post-activation performance enhancement
CTRL	Control condition
ISO	Isometric muscle action condition with total ICA duration of 27 s in 3 sets
T	Group consisting of trained participants
HT	Group consisting of highly trained participants
JH	Jump height
PP	Relative peak power output
RSImod	Modified reactive strength index
